# Organoid–microglia system for modeling the immune microenvironment of the brain and retina

**DOI:** 10.3389/fimmu.2026.1747589

**Published:** 2026-03-12

**Authors:** Jingjing Yu, Binxin Tang, Zhanjing Gu, Guanyuan Wang, Aijing Liu

**Affiliations:** 1 Department of Rheumatology and Immunology, The Second Hospital of Hebei Medical University, Shijiazhuang, Hebei, China; 2 Hebei Medical University-National University of Ireland Galway Stem Cell Research Center, Hebei Medical University, Shijiazhuang, Hebei, China; 3 Hebei International Joint Research Center on Rheumatic Diseases, Shijiazhuang, Hebei, China; 4 School of Biology and Engineering, Guizhou Medical University, Guiyang, Guizhou, China; 5 Hebei Research Center for Stem Cell Medical Translational Engineering, Shijiazhuang, Hebei, China

**Keywords:** microglia, organoids, neuroinflammation, stem cells, neuroimmune interactions, disease modeling, drug screening

## Abstract

Glial cells play a critical role in neural development, function, and immune regulation, with microglia serving as the principal immune cells of the central nervous system and retina. Although microglia are central to neuroinflammation and disease progression, progress in understanding human microglial biology has been limited by the lack of physiologically relevant *in vitro* models. Stem cell–derived brain and retinal organoids provide three-dimensional systems that recapitulate human tissue architecture and developmental trajectories, offering new opportunities to study neuroimmune interactions. This review summarizes strategies for integrating microglia into neural organoids through co-differentiation and transplantation, and outlines methodologies for establishing humanized immune microenvironments and assessing microglial maturation, migration, phagocytic function, and inflammatory activation. We highlight applications of organoid–microglia models in neurodevelopmental and neurodegenerative disorders, including autism spectrum disorder, Alzheimer’s disease, and retinal diseases, as well as their potential in drug screening and microglia-targeted interventions. Additionally, emerging technologies—such as organ-on-a-chip platforms, spatial transcriptomics, and multi-omics analyses—are enhancing the physiological relevance and analytical power of these systems. Overall, organoid–microglia platforms bridge a critical gap between conventional cell culture and *in vivo* models, enabling deeper insights into neuroimmune interactions and accelerating the development of precise immunomodulatory therapies.

## 1 Introduction

Glial cells are the major non-neuronal cell type in both the central (CNS) and peripheral nervous systems (PNS). Historically considered as passive supporters of neurons, glial cells are now recognized as active participants in brain development, function, repair, and immune responses (1, 2). Today, while neurons serve as the core units for information processing and transmission, glial cells are essential regulators of neural circuitry and information integration.

**Figure 1:**
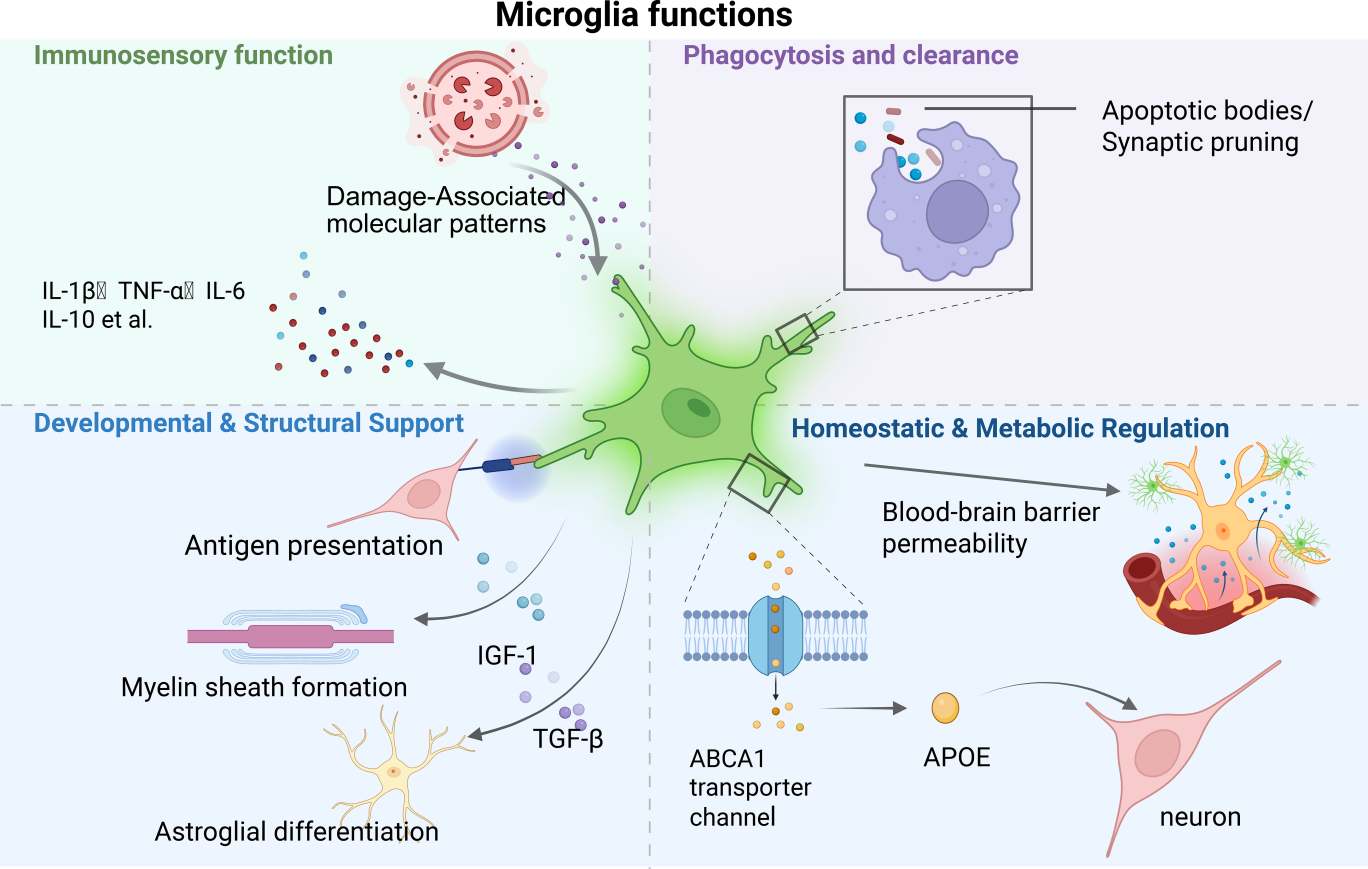
Microglial functions in the CNS. Schematic illustration of the multi-faceted functions of microglia in the CNS. The upper panels illustrate immunosensory functions: detection of damage-associated molecular patterns (DAMPs) and secretion of cytokines. Phagocytic and clearance: synaptic pruning and removal of apoptotic bodies. The lower panels depict: (Left) Developmental and structural support: microglia secrete factors such as insulin-like growth factor (IGF)-1 and transforming growth factor (TGF)-β to promote myelination and glial differentiation; (Right) Homeostatic and metabolic regulation: microglial monitoring of vascular integrity and regulation of lipid metabolism via ABCA1/APOE pathway. In the BBB component, astrocytes (yellow) provide structural support with their endfeet ensheathing the vasculature, while microglia (green) dynamically survey the neurovascular unit.

There are three major types of glial cells in the CNS: astrocytes, oligodendrocytes, and microglia (3). Astrocytes, the most abundant glial cell type (4), provide metabolic and nutritional support to neurons and regulate synapse formation, neuronal function, neurovascular coupling, blood flow, and homeostasis (5–7). Oligodendrocytes originate from neural progenitor cells in the ventricular zone and form myelin sheaths around axons during development (8). Microglia, the only glial cells of immune origin in the CNS, arise from erythro-myeloid progenitor (EMP) cells in the yolk sac. These EMPs differentiate into primitive macrophages, migrate into the developing neural tube, and ultimately give rise to microglia (9, 10). Microglia play unique roles in neuroinflammation, neuronal precursor regulation, and prenatal circuit establishment in both the brain and retina (**Figure 1**) (11–16). Their dysfunction is closely linked to neurodegenerative and neurodevelopmental disorders, including Alzheimer’s disease (AD), amyotrophic lateral sclerosis (ALS), and autism spectrum disorder (ASD) (17–22). However, traditional *in vitro* models fail to fully recapitulate these interactions due to the rapid transcriptomic drift of isolated microglia (23). Organoid technology offers a robust 3D platform to overcome the limitations of 2D culture in mimicking human-specific cellular architecture and signaling (24, 25). By integrating induced pluripotent stem cell (iPSC)-derived microglia, brain organoids (BOs) and retinal organoids (ROs) provide a powerful system for modeling the neuroimmune microenvironment in both health and disease. Furthermore, although microglia are the only myeloid-derived resident immune cells in the CNS, astrocytes also maintain the integrity of the blood-brain barrier and secrete various cytokines and chemokines. Microglia coordinate interactions among neurons, while astrocytes regulate neuronal activity through pruning (26).

**Figure 2:**
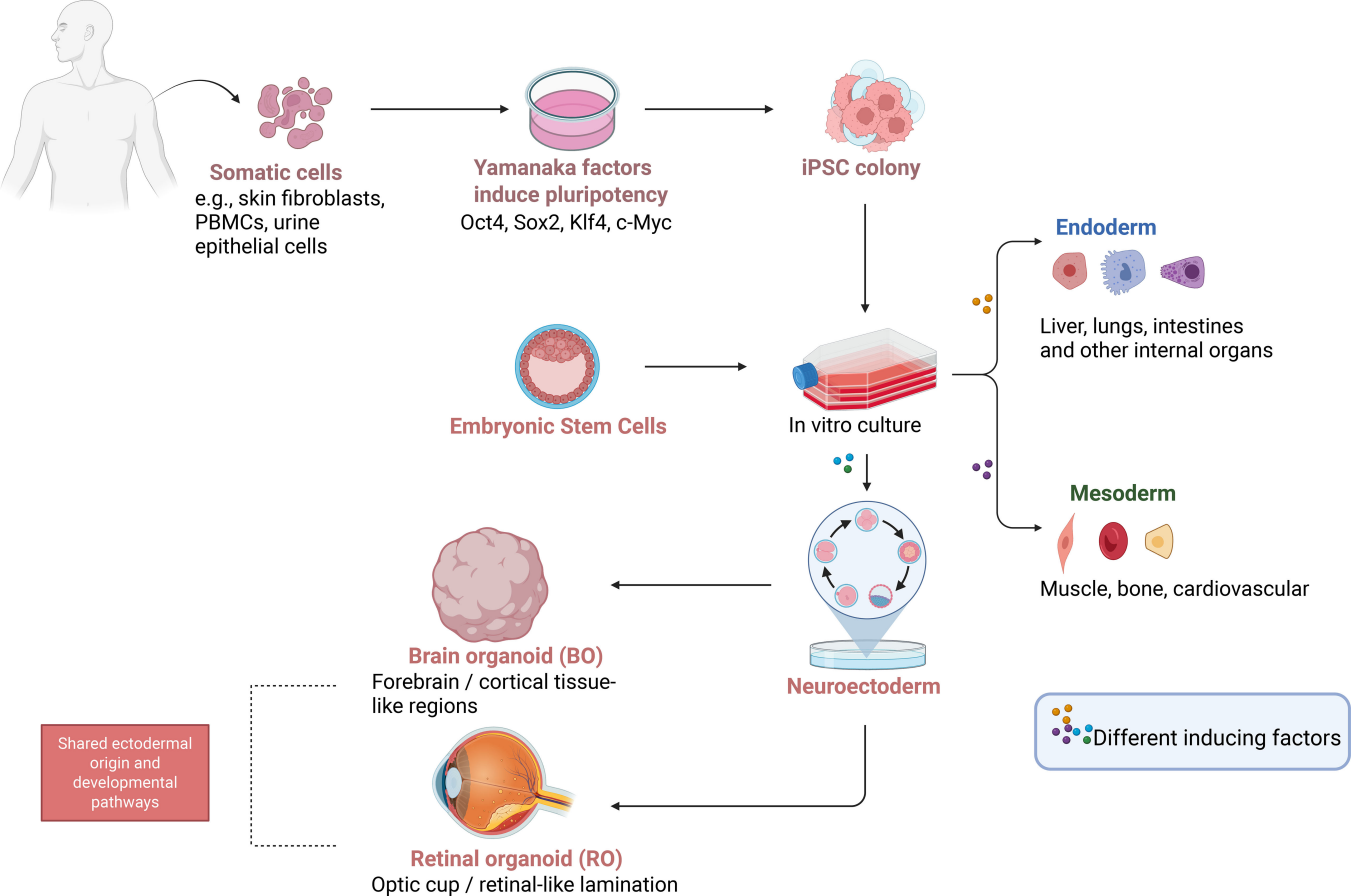
Derivation of brain and retinal organoids from pluripotent stem cells. Schematic illustration showing the generation of BOs and ROs from pluripotent stem cells. Somatic cells (e.g., skin fibroblasts, peripheral blood mononuclear cells (PBMCs), urine epithelial cells) are reprogrammed into iPSCs using Yamanaka factors (Oct4, Sox2, Klf4, c-Myc). iPSCs or ESCs are then cultured and guided to differentiate into three germ layers. Through neuroectodermal induction, ectoderm-derived progenitors develop into either BOs-, modeling forebrain/cortical-like regions, or ROs, forming optic cup–like retinal structures. Both organoid types share a common ectodermal origin and early developmental pathways, thereby providing human-specific 3D systems for modeling CNS and visual system development.

Integrated BO and RO models recapitulate early CNS and visual system development, providing a unique humanized neuroimmune microenvironment for studying neural development and disease mechanisms (**Figure 2**). Despite their potential, challenges such as optimizing integration timing, standardizing functional assays, and ensuring long-term stability remain to be addressed (27). This review systematically summarizes current strategies for integrating microglia with organoid systems and evaluates their functional impact. We further explore the applications of these platforms in modeling neurodevelopmental and neurodegenerative diseases. Finally, we highlight the transformative role of emerging technologies, including spatial transcriptomics and organ-on-a-chip systems, in addressing current modeling gaps and accelerating therapeutic discovery and screening.

## 2 Fundamentals

Microglia play a central role in the immune landscape of the CNS and are key players in various neurodegenerative diseases. In AD, blood–brain barrier disruption allows peripheral immune infiltration, while microglia respond by clearing amyloid-beta (Aβ) plaques and releasing pro-inflammatory cytokines such as tumor necrosis factor (TNF)-α and Interleukin (IL)-1β, thereby contributing to neuroinflammation. In Parkinson’s disease (PD), microglia phagocytose α-synuclein aggregates, potentially exacerbating dopaminergic neuronal damage (28, 29). In ALS, microglia adopt disease-associated phenotypes that promote inflammation and neuronal injury. Interestingly, microglia can also exhibit neuroprotective roles under specific pathological conditions (30). This duality reflects the functional plasticity of microglia, which are often described as existing along a spectrum between M1-like (pro-inflammatory) and M2-like (immunoregulatory) activation states (31).

As a peripheral extension of the CNS, the retina provides an additional system for exploring microglial immune regulation. The retina is an immune-privileged tissue, protected by the blood–retina barrier (BRB) and various intrinsic mechanisms. Under homeostatic conditions, retinal microglia support neuronal maintenance, synaptic pruning, and immune surveillance (32). Upon injury, infection, or ischemia, BRB disruption leads to peripheral immune infiltration. Retinal microglia rapidly activate, initially adopting a pro-inflammatory phenotype to contain damage, then transitioning to a reparative state to restore homeostasis (33). This dynamic response profile highlights microglia as essential regulators of retinal health. Dysregulated microglial activity is implicated in ocular diseases such as glaucoma, age-related macular degeneration (AMD), retinitis pigmentosa, and diabetic retinopathy (34). In these conditions, microglia contribute to neuronal degeneration through oxidative stress, inflammatory cytokine release, or aberrant synaptic pruning.

The term “humanized immune microenvironment” refers to a functional microenvironment constructed by human immune cells, encompassing their phenotypes, activation states, cytokine profiles, and interactions with surrounding tissues. In the CNS, microglia are the primary resident immune cells that shape local immune landscapes. Integrating microglia into BOs and ROs offers a promising strategy to model these interactions. BOs are particularly suited for studying neural development, neuro-inflammation, and diseases such as ASD, AD, and PD, while ROs enable exploration of immune privilege breakdown and microglial roles in ocular pathologies. These co-culture systems bridge the gap between 2D cultures and animal models, thereby enhancing disease modeling, drug discovery, and precision medicine approaches.

## 3 Methodological framework

### 3.1 Exogenous addition method

The exogenous addition method is currently the most widely applied strategy for organoid–microglia co-culture. This approach involves differentiating microglia from human induced pluripotent stem cells (iPSCs) or embryonic stem cells (ESCs) *in vitro* and introducing them into preformed organoids to create a three-dimensional neural microenvironment that better mimics physiological conditions. The optimal time point for microglia addition is typically when neuronal networks have formed but glial populations remain underdeveloped, allowing exogenous microglia to integrate into the organoid and modulate synaptic remodeling and neuronal excitability (35) Various delivery methods have been developed, including direct pipetting, embedding with matrix, centrifugation-assisted adhesion, and precision injection using microfluidic systems (36, 37). To enhance microglial viability and preserve their ramified morphology, cytokines such as IL-34, TGF-β1, and Macrophage colony-stimulating factor (M-CSF) are commonly supplemented (38, 39). Some studies have further supported microglial integration by adding fibroblast growth factor, epidermal growth factor, and heparin to brain organoids. In ALS and Parkinson’s disease-dementia models, researchers observed altered M1/M2 polarization and impaired phagocytic capacity. In AD models, microglia upregulated genes related to cholesterol transport and used high-density lipoprotein (HDL)-like particles to deliver cholesterol to neurons, thereby promoting neurogenesis (40). These findings confirm the feasibility and utility of exogenous addition method for modeling the immune microenvironment in neurodegenerative diseases.

This strategy is also applicable to ROs. Studies have shown that exogenous human iPSC-derived microglia can integrate into mature ROs, localizing near photoreceptors and ganglion cells with tissue-specific distribution and activation states (41). Upon inflammatory stimulation, these cells demonstrate phagocytic capabilities and M1/M2 polarization patterns consistent with *in vivo* microglia (42), making this model suitable for studying diabetic retinopathy and age-related macular degeneration (43). Moreover, microglia have shown compatibility with ROs from different donors, supporting the investigation of genetic heterogeneity and individual disease susceptibility.

In summary, the exogenous addition method is simple, time-efficient, and highly controllable, making it ideal for rapid modeling and drug screening. However, a key limitation is that microglia tend to remain on the organoid surface and fail to penetrate deeper layers, reducing their capacity for sustained interaction with neuronal circuits and long-term maintenance of homeostasis.

### 3.2 Spontaneous co-differentiation method

The spontaneous co-differentiation method allows microglial lineage cells to arise endogenously within brain organoids during their induction and development, without requiring external addition or transplantation. This strategy recapitulates early embryonic interactions between the mesoderm and ectoderm, enabling iPSCs to give rise to microglia-like cells alongside neurons and astrocytes, thereby establishing a more physiologically relevant 3D neuroimmune network.

Ormel et al. were the first to report the spontaneous emergence of microglia in human cortical brain organoids. These cells exhibited key functional characteristics, including migration, phagocytosis, and inflammatory responses, confirming the potential of brain organoids to intrinsically generate innate immune components (44). Subsequently, researchers refined protocols to reduce neuroectodermal stimuli and delay extracellular matrix coating, thereby facilitating the development of brain organoid-derived microglia (oMGs). These oMGs express myeloid transcription factors, canonical microglial markers, and the lysosomal protein CD68, and their transcriptomic profiles closely resemble those of human primary microglia. Compared to exogenously introduced microglia, oMGs demonstrate higher levels of integration and developmental synchrony within the organoid, allowing for reciprocal cell–cell interactions and more faithful modeling of CNS microenvironments. One comparative study showed that spontaneously differentiated microglia respond more efficiently to Aβ exposure, exhibiting enhanced clearance and tighter inflammatory regulation (45), suggesting greater biological relevance for modeling AD and other neurodegenerative conditions.

Spontaneous co-differentiation approaches have also been extended to ROs. In a novel 3D model, researchers co-cultured ROs with human iPSC-derived macrophage progenitor cells (MPCs), which migrated into the equivalent of the outer plexiform layer and established residency—mirroring microglial colonization patterns in the healthy retina. Over time, MPCs matured into ramified microglia-like cells with compact somas and long processes, morphological features typically observed only *in vivo*. Notably, during their maturation, these cells transitioned from a transient activation phase to a stable homeostatic state, marked by downregulation of pro-inflammatory genes and upregulation of anti-inflammatory genes. This co-culture system offers a powerful tool for studying microglial roles in retinal disorders and for conducting drug screening in a human-relevant setting (46).

Despite its strengths in cellular integration and developmental fidelity, this method presents several limitations. First, spontaneous microglial differentiation is susceptible to batch effects across iPSC lines and culture conditions, reducing reproducibility and controllability. Second, it does not allow for targeted introduction of patient-specific mutations, limiting its application in disease modeling and personalized medicine. Furthermore, due to the absence of embryonic niche factors, the resulting microglia may remain in a partially immature or unstable state (47). Wenzel et al. demonstrated that while microglia in innately differentiated unguided organoids exhibit superior Aβ clearance, their limited controllability and challenges in gene editing constrain their utility for large-scale disease modeling (45). Another critical drawback is that these models still lack vascular structures and crosstalk with non-neural embryonic tissues, which are essential for fully recapitulating *in vivo* brain development (38).

### 3.3 Chimeric transplantation approach

Human neural or microglial progenitor cells transplanted into the adult brain or spinal cord of rodents during the neonatal stage can widely engraft and functionally integrate with the host nervous system, forming highly chimeric human-mouse models. These models provide a powerful tool to study human microglial development and pathophysiology in a mature and intact physiological environment. Microglia are among the most difficult endogenous cell types to maintain in a mature state under traditional *in vitro* cerebral organoid culture. To overcome this limitation, researchers have developed chimeric transplantation models, in which human microglia or microglia-containing organoids are transplanted into mouse brains, allowing the host microenvironment to promote microglial maturation and functional integration (48). This method provides microglia with a vascularized, immunocompetent, and dynamically regulated environment, more closely mimicking their behavior in the human CNS (49, 50). One xenogeneic chimeric strategy involves differentiating human iPSCs into microglial precursors along a yolk sac developmental trajectory, promoting maturation with factors such as M-CSF, IL-34, and TGF-β1, co-culturing them with cerebral organoids, and finally transplanting the entire construct into mouse brains (51). Post-transplantation, the tissue receives host vascular support, maintains long-term survival, and continues expressing homeostatic microglial markers. Compared with injecting microglia alone, this strategy better preserves human-specific morphology and function, consistent with findings from Tomasz et al. (52). A major breakthrough of this model is its application in ASD, showing that microglia within the chimeric system exhibit morphology and transcriptomic profiles highly consistent with postmortem patient brains, largely driven by the organoid microenvironment rather than intrinsic cell properties, indicating its ability to capture disease-relevant neuroimmune phenotypes.

Recently, chimeric transplantation strategies have been extended to RO systems. Studies show that human iPSC-derived microglia injected beneath the mouse retina can migrate into neural retinal layers, survive long-term, exhibit ramified morphology, and express homeostatic markers such as IBA1 and hP2RY12 (53), providing a valuable tool to model retinal immune microenvironments. Current retinal models mainly include two approaches (1): integrating microglia into ROs *in vitro* to observe morphological maturation and immune functions; and (2) injecting human iPSC-derived microglia into live mouse retina for long-term integration and functional expression. Both approaches demonstrate the capacity of microglia to assimilate into retinal tissue and maintain homeostatic functions in the retinal microenvironment. However, no studies have yet transplanted a complete “RO + microglia” construct into animals to generate fully chimeric models. Future strategies may further enhance physiological fidelity and facilitate studies of retinal neuroimmune interactions.

Chimeric transplantation offers several advantages. In both brain and retinal regions, the host environment provides vascular support and dynamic metabolic inputs, maintaining microglial homeostasis, enhancing migration and ramified morphology, and closely approximating *in situ* neuroimmune interactions (52). Notably, microglia in cerebral organoid chimeras display disease-specific transcriptomic features, and in the retina, they can integrate and survive long-term, reflecting near *in vivo* morphology and function. Limitations include dependence on immunodeficient animal models, technical complexity, and the need for long-term dynamic monitoring, which require further optimization for broader applicability. To provide a clearer overview of the current landscape, a comprehensive comparison of three organoid and glial cell modeling strategies is presented in **Table 1**.

**Table 1:** Comprehensive comparison of three organoid and glial cell modeling strategies.

Comparison dimensions	Exogenous addition	Co-differentiation	Chimeric transplantation
Microglial lineage	iMG/Primary	Spontaneous co-differentiation	iMG transplanted and relocated
Maturity	Moderate, slightly activated	Low to Medium	Highly mature, approaching *in vivo*
Simulation realism	Organoids	Organoids	Brain Environment
Technical Difficulty	Low	Medium	High
Experimental cycle	Short	Medium	Long
Application Focus	High-throughput drug screening, acute inflammatory response simulation.	Simulating early development, the initial stage of neuroimmune interactions	Investigate glial cell migration across regions (such as from non-neural to neural areas), barrier interactions.

iMG, induced Microglia-like cells.

**Figure 3:**
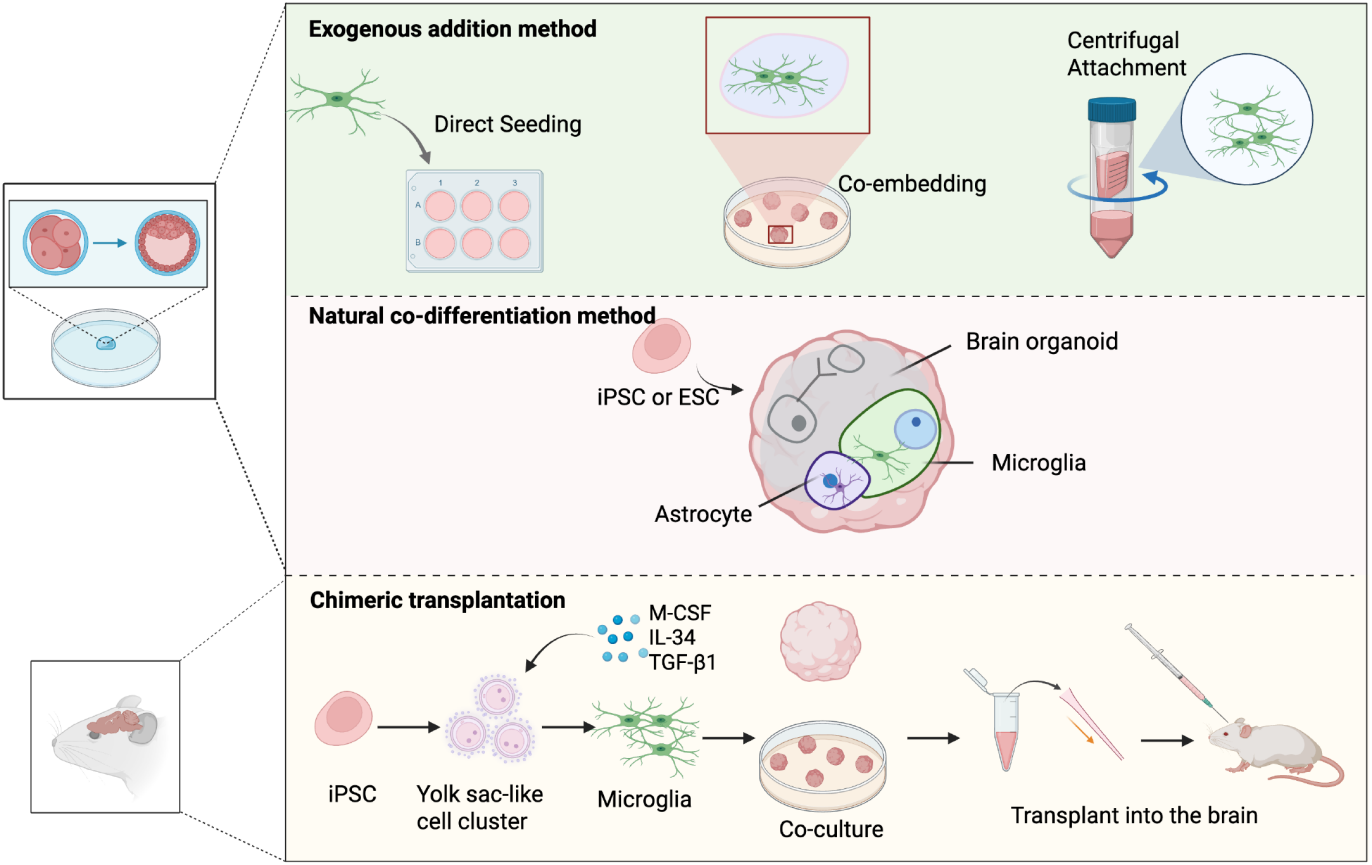
Strategies for integrating microglia into organoid models. Schematic illustration of three representative approaches for constructing organoid–microglia systems. Exogenous addition—microglia are externally introduced into preformed organoids via direct seeding, co-embedding, or centrifugal attachment. Spontaneous co-differentiation—microglia and neural cells co-develop from iPSCs or ESCs within the same culture, mimicking *in vivo* ontogeny. Chimeric transplantation—iPSC-derived microglia are co-cultured with organoids or transplanted into mouse brains to study their maturation and functional integration.

In summary (**Figure 3**), the strategy selection should be guided by specific research objectives: the co-differentiation approach is preferred for investigating the early developmental roles and neuroimmune functions of microglia. Exogenous addition, however, offers a more modular and high-throughput solution suitable for drug efficacy testing and acute neuroinflammation studies. For those focusing on directed cell migration or regional interactions, chimeric transplantation provides an optimal spatial framework for observing dynamic cell recruitment and integration processes.

## 4 Disease modeling and immune dynamics

### 4.1 Neurodevelopment and microglial function

Microglia are the primary resident immune cells of the CNS, originating from yolk sac mesodermal precursors and colonizing the CNS around gestational week 5 (54). In adults, microglia continuously survey the brain parenchyma, regulating synapse formation, pruning, and remodeling, while also influencing neurotransmitter release, synaptic plasticity, and overall circuit development (55). Their processes interact extensively with neurons, astrocytes, oligodendrocytes, and the vasculature, contributing to immune surveillance, debris clearance, and homeostatic regulation (56). With the advent of iPSC technology, human microglia-like immune cells can be generated *in vitro* and co-cultured with cerebral organoids, reproducing amoeboid morphology, migratory behavior, and responses to local neural damage, such as chemotaxis, phagocytosis, and immune activation (57). Integrated microglia can modulate neural progenitor transcription and influence synaptogenesis, recapitulating early neuroimmune interactions and providing high-fidelity models for studying human brain development and disease (52).

In the visual system, the retina, as an extension of the CNS, exhibits a complex neural network and unique immune features. Single-cell RNA sequencing has identified four retinal microglia (RMG) subtypes—RMG0, RMG1, RMG2, and RMG3—revealing their functional heterogeneity: RMG0 and RMG2 are primarily associated with homeostasis; RMG1 is enriched in oxygen-induced retinopathy and mediates pro-inflammatory responses; RMG3 is linked to angiogenic pathways, potentially involved in pathological or reparative neovascularization. These findings highlight the dynamic and region-specific roles of retinal microglia.

Long-term culture techniques are crucial for simulating microglia maturation and their transition to an aged state. The air-liquid interface cerebral organoid (ALI-CO) technology, developed by Giandomenico et al., optimizes nutrient and oxygen delivery, extending unguided organoid survival for up to one year and providing a critical window for studying late-stage human neurodevelopment (58). In their latest study, Rittenhouse et al. demonstrated that microglia within guided cortical organoid-based microphysiological systems spontaneously transition to a ramified homeostatic state over 9 weeks, significantly promoting neuronal electrophysiological maturation. This transition from an “embryonic” to an “adult steady-state” condition is an indispensable prerequisite for studying pathological aging (59). Establishing functionally mature organoid models is a biological prerequisite for simulating pathological aging. To overcome the widespread “juvenile” limitations of current organoid models, researchers have begun superimposing specific aging stressors onto long-term cultured mature systems. Genetic approaches have been employed to induce senescence-associated protein expression to mimic degenerative phenotypes such as nuclear envelope abnormalities (60). Biochemical approaches simulating Aβ deposition or chronic inflammatory challenges have effectively reconstructed the neuroimmune microenvironment observed in diseases like AD (45). These innovative methods enable precise capture of the critical transition points at which human-derived microglia shift from homeostatic surveillance to aging-associated secretory phenotypes. This holds significant research value for elucidating the failure of neuroimmune interaction networks in aging or neurodegenerative disease contexts.

### 4.2 ASD modeling and mechanistic studies

ASD is characterized by social communication deficits and repetitive behaviors, with growing evidence linking its pathogenesis to neuroimmune dysregulation. Early inflammatory insults, including maternal immune activation (MIA) and childhood infections, are critical environmental risk factors (61). MIA can over-activate fetal microglia, triggering chronic inflammation and ASD-like behaviors, disrupting synaptic pruning and neurogenesis, and impairing neuronal maturation (62). Microglia are pivotal in this process, as their over-activation can disrupt synaptic pruning and neurogenesis (63, 64). Recent advancements in human cerebral organoid–microglia models have provided critical insights into these mechanisms. For instance, studies have demonstrated that microglia-mediated aberrant synaptic pruning is a key driver of ASD pathology, as observed in gene-deficient organoid models where excessive pruning led to reduced synaptic transmission (65). Furthermore, experiments using neural–immune organoid chimeras (iHBO) have emphasized that the ASD microenvironment, rather than intrinsic genetic factors, predominantly dictates the reactive morphological changes in microglia (51). These findings underscore the profound influence of the niche microenvironment on immune cell behavior in ASD.

### 4.3 AD modeling and mechanistic studies

AD is characterized by Aβ plaque accumulation, Tau hyperphosphorylation, and neuroinflammation. Microglia play dual roles in this process, encompassing both: protective phagocytosis and chronic inflammatory activation (57). While traditional models are limited by species differences, integrating microglia into human cerebral organoids enables the recapitulation of complex pathologies, including responses to Aβ oligomers, chemotaxis, and cytokine secretion (57, 66). Recently, organoid models have been instrumental in deciphering the impact of genetic variants on microglial function. For instance, studies using iPSC-derived AD models identified that the *APOE3* Christchurch mutation enhances microglial resistance to lipid peroxidation and promotes phosphorylated Tau (pTau) clearance, thereby preserving neuronal network integrity (67). These findings not only provide insights into *APOE*-targeted therapeutic strategies but also demonstrate the utility of organoid platforms for screening compounds that shift microglia from pro-inflammatory to neuroprotective phenotypes.

### 4.4 Retinal organoid–microglia applications

As an extension of the CNS, the retina exhibits a highly stratified neuronal network and a tightly regulated immune microenvironment. Microglia play a crucial role in maintaining normal retinal vascular development. They facilitate the connection of neighboring sprouts and promote vascular anastomosis, thereby contributing to the formation of a robust vascular network. Moreover, microglia guide the directional growth of developing vessels, and their displacement results in aberrant vascular patterning (68). In addition to promoting neovascular development, microglia also exert anti-angiogenic effects to maintain vascular homeostasis. Vascular endothelial growth factor (VEGF)-C–positive microglia located at vessel anastomosis sites activate VEGFR-3 signaling in endothelial tip cells, leading to the induction of Notch target genes and reduced sensitivity to VEGF gradients. This mechanism effectively prevents excessive angiogenesis (69). Nobuhiko Shiraki et al. identified PAX6-positive microglia-like cells within human iPSC-derived ocular organoids, which exhibited features of yolk sac–derived myeloid progenitors and immune surveillance capability, closely resembling primitive microglial morphology. This was the first observation of endogenously generated microglia-like cells in ocular organoids, providing a foundation for reconstructing retinal immune mechanisms *in vitro (*70). Subsequently, Ayumi et al. developed a “RO + hiPSC-derived MPC” co-culture model, achieving microglial integration with ramified morphology, dynamic cytokine transitions from activation to homeostasis, and homeostatic transcriptomic profiles (46). Disease-associated mutations or stressors can be applied to study microglial activation, migration, phagocytosis, and neuroimmune interactions. Patient-specific microglia carry genetic mutations can be engineered via CRISPR to examine disease-relevant phenotypes (42). To ensure that integrated microglia within ROs possess true functional fidelity rather than mere morphological coexistence, researchers have established a suite of validation assays. **Table 2** summarizes the key functional assessments used to evaluate microglial activity in RO systems, highlighting their phagocytic competence, chemotactic migration, and immunological responsiveness.

**Table 2:** Key functional assays for microglial activity in RO.

Function	Assay method	Observed phenotypes
Phagocytosis	Incubation with pHrodo-labeled particles or apoptotic photoreceptor debris	Internalization of fluorescence signals; reduction of debris in the ONL
Chemotactic Migration	Localized laser-induced injury or ATP micro-gradient application	Rapid recruitment of microglia somata and processes to the injury site
Immune Surveillance	Time-lapse live-cell imaging	Continuous, dynamic extension and retraction of microglial processes
Inflammatory Response	Stimulation with high glucose (DR model) or pro-inflammatory cytokines	Upregulation of TNF-α, IL-1β, and shift to amoeboid morphology

### 4.5 Other neurodegenerative diseases

Astrocytes are the main cholesterol-producing cells in the adult brain and are crucial for neurite outgrowth and synaptogenesis (71). Martina Absinta’s team created 3D organoids containing microglia and astrocytes, recapitulating typical neuroinflammation and demyelination observed in multiple sclerosis (MS) (72). This “immune–glia–neuron” tri-culture strategy is also applicable to other neurodegenerative disease models (such as ALS and PD).

In ALS, microglia are thought to contribute to motor neuron injury, whereas in chronic pain, they exhibit surprising sex-dependent differences (73). Traditional co-cultures often suffer from batch effects and lack physiological complexity; thus, integrated tri-culture organoids (comprising neurons, astrocytes, and microglia) have emerged to better recapitulate the “immune–glia–neuron” axis in diseases like MS (74). These models are particularly transformative for rare disorders such as Hereditary diffuse leukoencephalopathy with axonal spheroids (HDLS), where patient-derived organoids revealed that CSF-1R CSF1R mutations mutations disrupt microglial homeostasis as early as the fetal stage. This disruption leads to adult upregulation IL-1β, identifying IL-1β inhibitors as potential targeted therapies (75). Furthermore, microglia-integrated organoids have been utilized to study immune responses under extreme conditions, such as microgravity during spaceflight, which was found to modulate neuronal maturation and proliferation genes (76). Together, these applications demonstrate the versatility of organoid platforms in modeling diverse pathological and environmental stressors.

Although previous studies have demonstrated that microglia can arise spontaneously within brain organoids or coexist with neurons via exogenous addition or chimeric transplantation, current organoid–immune system models still face significant limitations in terms of cellular composition stability, the physiological relevance of spatial organization, and functional coupling. To further enhance the accuracy and translational potential of organoid models for recapitulating human CNS–immune interactions, the integration of advanced bioengineering techniques and multidimensional regulatory strategies is urgently needed, offering promising avenues for future breakthroughs in this field.

## 5 Cutting-edge engineering

### 5.1 Microfluidic chips (organ-on-a-chip)

Microfluidic chips, as integrated and high-precision three-dimensional culture platforms, enable the development of novel systems designed to mimic the complex architecture and physiological perfusion of target organs. The lack of a vasculature in organoids has long been considered a major limitation for accurately recapitulating the *in vivo* environment. Microfluidic systems can deliver fluids precisely through microchannels, facilitating nutrient supply, metabolic waste removal, and dynamic cell-environment interactions. This approach effectively addresses challenges associated with conventional culture methods, including nutrient insufficiency, loss of polarized structures, and difficulties in immune cell integration, providing a feasible strategy for constructing high-fidelity organoid–immune cell co-culture models.

In BOs, microfluidic chips have been shown to promote neuronal polarity, long-term survival, and the maintenance of neural electrical activity. Various “brain-on-a-chip” systems have been developed, including axon-guidance chips (77), blood–brain chips (78), and neurovascular-unit-on-a-chip platforms (79), and have been widely applied in studies of brain tumors, vascular disorders, brain injury, and neuroinflammation. Compared with conventional static culture methods, these brain-on-a-chip systems provide higher physiological fidelity. Zheng Ao and colleagues (80) developed a tubular brain-on-a-chip platform that directs the differentiation of neural progenitor cells along microchannels to form centrally organized structures with uniform polarity, enhancing tissue viability and perfusion efficiency while optimizing conditions for microglial migration and observation. This design improves neuronal polarity consistency and, when combined with a rocking perfusion system, allows controlled flow rates to provide stable nutrient supply, further enhancing tissue survival. Brain-on-a-chip systems can also incorporate blood–brain barrier models for drug permeability assessment and the study of immune regulatory mechanisms (81), demonstrating significant value for early-stage screening and mechanistic research in complex diseases such as AD (82). Furthermore, microfluidic devices integrated with 3D printing technology enable the construction of organoids with more uniform structure and higher stability, allowing cultures to be maintained for over 10 days without manual intervention, thereby significantly reducing handling frequency and contamination risk (83).

Similar microfluidic strategies are now being extended to the visual system by integrating ROs with microfluidic platforms to establish more physiologically relevant retina-on-a-chip models. This platform allows precise control of fluid shear stress and oxygen distribution within human ESC-derived RO cultures, thereby promoting retinal progenitor cell expansion, regulating the differentiation and axonal growth of retinal ganglion cells, and providing a controlled and stable microenvironment for efficient maturation (84). Furthermore, microfluidic platforms can finely tune the fluidic environment to improve co-culture conditions of ROs and retinal pigment epithelial (RPE) cells, enhancing cell polarity and differentiation while promoting extracellular matrix (ECM) synthesis and functional maturation, significantly increasing the physiological relevance of the organoids. In a study using an RO-RPE co-culture model derived from iPSCs of a *USH2A*-mutant patient, the microfluidic chip facilitated the elucidation of key molecular mechanisms underlying retinitis pigmentosa, such as PI3K-Akt pathway inactivation and weakened ECM–receptor interactions. Additionally, the system’s automated handling capability highlights its broad potential as a platform for studying retinal degenerative diseases (85).

### 5.2 Spatial transcriptomics and multi-omics analysis

Spatial transcriptomics (ST) is a high-throughput technique that integrates tissue spatial information with transcriptional profiles, enabling precise mapping of specific cell types within tissues and characterization of their gene expression patterns (86). Organoids, as complex three-dimensional microtissue models, are relatively small, with a limited number of cells in any given cross-section—making it challenging for conventional characterization methods to fully capture their internal spatial organization. In recent years, ST has been increasingly applied to brain organoid research to reveal patterns of cellular distribution and region-specific functional characteristics. In ST studies, cell type annotation often relies on large-scale single-cell RNA sequencing (scRNA-seq) or epigenetic datasets, which are subsequently projected onto ST data to achieve spatially resolved identification of cell identities (87, 88). The integration of lineage tracing with spatial transcriptomics effectively addresses the limitations of reconstructing developmental trajectories in traditional modeling. For instance, He et al. (89) successfully reconstructed the migration pathways of cells from the ventricular zone to cortical-like structures in unguided cerebral organoids using a “spatial lineage recording” technique. This approach overcomes the previous constraint of observing only the end-state of organoids, enabling researchers to precisely pinpoint when and where microglia become involved in neural circuit formation. Consequently, it provides crucial evidence for deciphering the spatiotemporal dynamics of neuroimmune interactions. Subsequently, standard ST methods were applied to investigate how the overall tissue morphology of unguided BOs influences developmental processes (90), demonstrating the significant potential of spatial gene expression analysis in organoid modeling. As reviewed by You et al, ST has been used to validate spatial associations between disease-associated microglia, amyloid plaques, and responses to traumatic brain injury (91). In parallel, researchers have combined the CRISPR/Cas9 system to embed editable barcode arrays into the cellular genome; through timed cleavage and insertion mutations, unique “lineage records” are generated during cell proliferation. These records can be read concurrently with scRNA-seq, providing both lineage and gene expression information (89). Applied to brain organoids, this technology enables reconstruction of developmental pathways from progenitor cells to neurons in cortical-like structure, revealing aberrant differentiation trajectories. When further integrated with ST, it allows comprehensive mapping from cellular lineage and migratory paths to spatial localization and functional states, establishing a full-chain analytic framework.

Studies have shown that microglia exhibit pronounced transcriptional heterogeneity across different brain regions, such as the cerebral cortex, hippocampus, cerebellum, and striatum. For example, microglia in the cerebellum and hippocampus of mouse models display heightened immune vigilance and upregulate co-regulated gene clusters associated with energy metabolism (92). These regional differences underscore the ability of microglia to rapidly respond to local microenvironments, with their states displaying marked temporal and spatial dependency and exhibiting dynamic, reversible, and non-fixed functional characteristics. Traditional scRNA-seq can identify cell subpopulations, but due to the loss of critical anatomical context during tissue dissociation, it struggles to reveal physical interactions between microglia and their neighboring neurons or pathological structures such as Aβ plaques. The introduction of ST technology effectively addresses this limitation. It not only identifies cellular identities but also resolves how microglia undergo state transitions induced by local niche signals while preserving the tissue’s native structure. Therefore, integrating ST with scRNA-seq in brain organoids holds promise for constructing spatial transcriptomic maps of multiple cell types, indirectly revealing the spatial distribution and states of microglia-like cells, and providing critical technical support for modeling human neuro–immune development.

Current mainstream ST platforms, such as 10x Genomics Visium, typically capture regions with diameters of 50–100μm, often encompassing multiple cells. As a result, their spatial resolution is limited, making it challenging to precisely localize individual microglia and determine their states, particularly in densely populated or structurally complex brain regions. In contrast, *in situ* hybridization and sequencing techniques such as MERFISH (93), seqFISH (94), and FISSEQ (95) offer higher spatial resolution, enabling expression measurements at single-cell or even subcellular levels, although they remain constrained by lengthy image acquisition times and equipment complexity. Moreover, ST primarily captures mRNA expression signals and does not directly reflect protein expression or cellular functional behavior. Therefore, integration with immunohistochemistry, functional assays, and multi-omics approaches is necessary to enhance the accuracy of cellular state characterization and the biological interpretability of the data. By providing a conceptual shift from analyzing cell populations to mapping spatial networks, ST enables researchers to quantify the distribution density and activation thresholds of microglia across different functional zones within organoids. This breakthrough overcomes the previous scientific bottleneck of only being able to observe macroscopic immune responses without localizing the microscopic immune microenvironments.

### 5.3 Future perspectives and limitations

Co-culture models integrating organoids with microglia have made significant progress in recapitulating the human CNS immune microenvironment. Beyond the aforementioned advanced technologies, investigators have employed CRISPR to precisely edit core pathogenic genes in AD, such as *APP, PSEN1, PSEN2*, and *APOE*, with the potential to reduce Aβ deposition and mitigate disease progression (96). In addition to conventional knockout and overexpression strategies, recently developed non-homologous repair CRISPR editing approaches enable rapid introduction of targeted mutations or reporter genes in organoids, thereby enhancing the efficiency of constructing tissue-specific human models (97). As an *in vitro* research platform, organoids have been employed to establish fully human neuroimmune brain organoid models (e.g., using guided regional organoids to simulate diffuse midline glioma), providing powerful tools for deepening our understanding of organoid biology and for developing novel therapeutic strategies (98). The therapeutic potential of these models is increasingly evident in personalized medicine and high-throughput drug screening. Their core advantage lies in overcoming the limitations of traditional 2D cell lines or primary cultures, which lack spatial structure and cell-cell interactions, by providing a 3D microenvironment that more closely approximates human physiological conditions. Although therapeutic clinical trials involving direct transplantation of brain or retinal organoids remain in their infancy due to ethical and safety considerations (most studies registered on ClinicalTrials.gov are mechanistic in nature), their application as “clinical trials in a dish” is rapidly expanding. In the field of precision medicine, patient-derived organoids authentically replicate the molecular characteristics of individual tumors, providing a critical platform for exploring cancer progression mechanisms and assessing drug sensitivity (98). In neurodegenerative and retinal disease research, organoids have successfully characterized the pathological progression of genetic disorders such as Usher syndrome and Leber congenital amaurosis, serving as vital tools for validating gene therapy targets (e.g., interventions targeting *CEP290* or *USH2A* mutations) (99). Additionally, organoids demonstrate significant advantages in infectious disease modeling. For instance, brain organoids used to simulate Zika virus-induced microcephaly exhibit a reduced tissue volume phenotype that closely mirrors clinical pathological findings (100).

**Figure 4:**
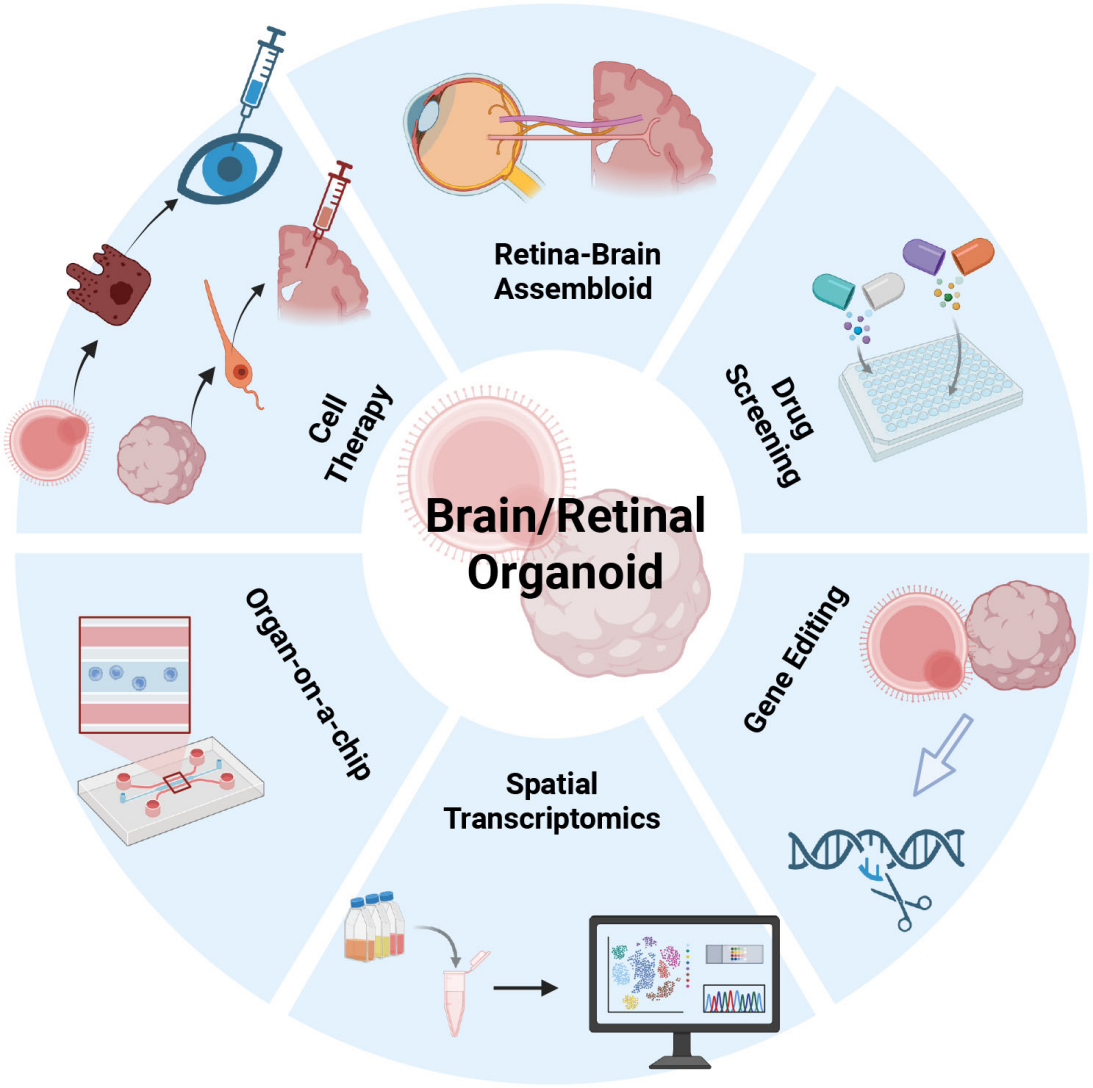
Frontier applications of retinal and brain organoids. Schematic illustration depicting key translational applications of retinal and brain organoid platforms. Retinal–brain assembloids enable the study of neural connectivity and visual pathway integration. Organoid-based drug screening platforms facilitate high-throughput evaluation of therapeutic compounds. Organoid-derived cells can also be employed in cell replacement strategies to repair or replace damaged neural tissues. Integration with organ-on-a-chip systems allows dynamic modeling of physiological microenvironments. Spatial transcriptomics provides insights into regional gene expression patterns, while gene editing technologies (e.g., CRISPR/Cas9) enable targeted manipulation for disease modeling and mechanistic studies.

Compared to animal models—which are limited by significant species differences—or adult ex vivo tissues—which suffer from limited availability and difficulties in dynamic experimentation—organoid models based on iPSCs demonstrate exceptional operational feasibility while maintaining clinical fidelity. As summarized in **Table 3**, organoids possess irreplaceable advantages in simulating human-specific spatiotemporal development and immune responses. In the future, with the maturation of vascularization techniques and long-term culture systems, organoid models will further enhance physiological fidelity. This progress will not only help adhere to the 3Rs principle (Replacement, Reduction, Refinement) and minimize reliance on animal experiments but also accelerate the precise translation of neuroimmunomodulatory therapies from the bench to the bedside (**Figure 4**).

**Table 3:** Comparative analysis of experimental models in neuro-immune and disease research.

Model	Source	Pros	Cons	Advantage
2D Cell Lines	Immortalized cells	Low cost; high-throughput screening; ease of maintenance.	Lack of 3D architecture; poor cellular heterogeneity; loss of cell-cell interactions.	--
Animal Models	Rodents, non-human primates	Complete physiological and systemic environment (e.g., blood circulation).	Significant species differences; low clinical translation rate; high ethical burden.	--
Ex Vivo Human Tissue	Surgery or cadaveric donors	Authentic human anatomical structure and pathology.	Limited sample availability; inability to perform long-term dynamic experiments.	--
Organoids	hiPSCs/Primary progenitors	Human genetic background; 3D spatial organization; recapitulates neuro-immune crosstalk; high manipulability.	Absence of functional vasculature; immature fetal-like states; batch-to-batch variability.	Bridges the species gap; supports personalized medicine (Clinical Trial in a Dish); aligns with 3Rs principles.

## 6 Conclusion

Although brain-on-a-chip technologies demonstrate considerable promise, they remain in the early stages of development and face several challenges, including a lack of standardized chip structures, insufficient coordination between organoids and the chip environment, and difficulties in maintaining stable immune cell populations. Current studies on retina-on-a-chip models have primarily focused on barrier integrity, electrophysiological function, and nutrient delivery, with limited attention given to modeling the local immune microenvironment. Microglia, as the key resident immune cells of the CNS, play critical roles in tissue homeostasis, inflammation regulation, and neural development. Although they have been successfully incorporated into BOs, no attempts have yet been made to integrate microglia into RO-chip systems. This gap limits our understanding of retina–immune interactions, particularly in inflammation-associated diseases such as age-related macular degeneration and diabetic retinopathy. In the future, introducing microglia or retinal resident immune cells into microfluidic RO systems could not only establish more complete models of the retinal immune microenvironment models but also provide powerful tools for investigating blood–retina barrier disruption, inflammation-mediated neurodegenerative mechanisms, and immunomodulatory drug screening. The integration of multidisciplinary technologies, including spatial transcriptomics, multimodal imaging, and programmable microfluidic systems, holds promise for addressing current challenges: high model heterogeneity, limited functional plasticity, and difficulties in standardization. Furthermore, advancing interconnected multi-organoid models (e.g., brain-retina-gut axis) could more accurately recapitulate the systemic pathogenesis of neurological diseases and expand their applications in drug screening, personalized therapy, and early-stage preclinical translation.
